# Patent *Troglostrongylus brevior-, Aelurostrongylus abstrusus-, Angiostrongylus* sp.-, and *Crenosoma* sp. infections in wild Eurasian lynxes (*Lynx lynx*) and their habitat-sharing gastropod intermediate hosts

**DOI:** 10.3389/fvets.2025.1515507

**Published:** 2025-07-03

**Authors:** Marcel Haas, Lisa Segeritz, Ole Anders, Tomma Lilli Middelhoff, Akary Myat Tun, Seyed Sajjad Hasheminasab, Berardino Cocchiararo, Alena Dusch, Anja Taubert, Carlos Hermosilla

**Affiliations:** ^1^Institute of Parasitology, Biomedical Research Center Seltersberg, Justus Liebig University Giessen, Giessen, Germany; ^2^Harz National Park, Wernigerode, Germany; ^3^Lplan – Planning Office for Landscape and Aquatic Ecology, Erlensee, Germany; ^4^Centre for Wildlife Genetics, Senckenberg Research Institute and Natural History Museum Frankfurt, Gelnhausen, Germany

**Keywords:** *Aelurostrongylus abstrusus*, *Angiostrongylus*, *Crenosoma*, Eurasian lynx, *Lynx lynx*, *Troglostrongylus brevior*, wildlife

## Abstract

The formerly widely spread Eurasian lynx (*Lynx lynx*) nowadays represents an endangered large wild felid species in Germany. Recent and ongoing conservation efforts have succeeded in establishing small but stable lynx populations in distinct parts of Germany. However, very little is known on the occurrence of neglected and re-emerging gastropod-borne cardiopulmonary nematodes in wild *L. lynx* populations in Europe. Therefore, the aim of current study was to estimate metastrongyloid infections in, a group of seven free-ranging, (sub-) adult Eurasian lynxes from the Harz Mountains (Germany) which were equipped with GPS/GSMS collars and in resident gastropod intermediate host populations. Both, lynx scat samples (*n* = 24) and terrestrial gastropods (*n* = 153) were collected in close proximity to prey remains left behind by Eurasian lynxes respectively in natural habitats in a non-invasive and un-molested manner. Fresh fecal samples were analyzed for the presence of metastrongyloid first-stage larvae (L1) by standard Baermann funnel technique and morphologically identified to genus level. Morphological metastrongyloid L1 were additionally investigated by PCR for final species identification. Terrestrial gastropods (i.e., slugs, semi-slugs, snails) were morphologically identified to genus level, thereafter artificially digested and analyzed for the presence of lungworm larvae. This work delivers a first report on the occurrence of patent *Troglostrongylus brevior-*, and *Crenosoma* sp.-infections in wild Eurasian lynxes in Germany and re-confirms recent findings on *Aelurostrongylus abstrusus-* and *Angiostrongylus* sp. infections in these lynxes. Overall, a total lungworm occurrence of 37,5% (9/24) was detected in assessed Eurasian lynx samples and 51.1% (4/7) of lynxes showed patent metastrongyloid infections. In digested terrestrial gastropods, 1.3% (2/153) contained *A. vasorum* larvae, underlining a successful propagation of *A. vasorum* life cycle in the Harz Mountains. Hence, we recommend regular monitoring for metastrongyloid infections not only in wild Eurasian lynxes but also in obligate intermediate hosts to better understand their impact on animal and population health to support current conservation efforts on this endangered large felid species in Europe.

## 1 Introduction

The Eurasian lynx (*Lynx lynx*) represents the largest felid apex predator in Europe and, in consequence, plays a fundamental role in the maintenance of ecosystem health by not only influencing food web composition but also increasing biodiversity in natural biomes (1–7). Consistently, as a natural apex predator, *L. lynx* might affect prey behavior, number and composition and thereby indirectly influencing flora and fauna biodiversity (5–7). Additionally, the surrounding of the remains of Eurasian lynx prey, also referred to as killing sites, can be recognized as such for several weeks, since lynx feed on their prey multiple times, depending on the prey size and eventual disturbances. Killing sites are nowadays considered as important micro-ecosystems influencing environmental biomes (5–8). Consistently, killing sites can provide valuable data on predator-prey relationships, on complex host-parasite interactions, and additionally serve as nutritional sources for numerous vertebrates, invertebrates and microbes (8–16). Despite a positive impact of wild Eurasian lynxes on ecosystem preservation, their crucial role in biodiversity improvement is generally underestimated in Germany and elsewhere (2–7).

Two hundred years ago, the geographic distribution of Eurasian lynxes ranged from the European mainland to Central Asia and from the Tibetan plateau of China to Eastern parts of Russia (17–22). The Eurasian lynx has the IUCN (International Union for the Conservation of Nature) status least concern and is strictly protected by the Convention on International Trade in Endangered Species of Wild Fauna and Flora (CITES), the Council Regulation (EC) 338/97 and Federal Species Protection Regulations (FSPR) in face of its population vulnerability (19, 21). However, according to the IUCN, the total Eurasian lynx population is listed as “least concern” since it shows a wide distribution in sparsely populated geographic areas of Eastern Europe and Asia (19, 21, 23). In contrast, in Germany, Eurasian lynxes are still facing extinction and are therefore listed as “critically endangered” according to the IUCN and the German Red List Centre (GRLC), based on their low individual numbers and critical population fragmentation (18–25). Main threats for *L. lynx* survival in Central Europe are poaching, traffic accidents, habitat fragmentation, habitat loss and insufficient presence of prey. Moreover, infectious diseases like viral infections (e.g., canine distemper, FeLV, FIV) and parasite infestations (e.g., *Sarcoptes*) negatively impact small Eurasian lynx populations as it is the case for the re-introduced Harz Mountain population (23, 26). Given that ecto- and endoparasitoses are well-known as causes of suffering and decline (27, 28), regular monitoring seems relevant for conservation issues. Accordingly, feline gastropod-borne metastrongyloid nematodes, such as *Aelurostrogylus abstrusus, Angiostrongylus chabaudi, Crenosoma vismani*, and *Troglostrongylus brevior*, can cause bronchopneumonia and cardiopulmonary disorders in various definite hosts, including wild felids and lynxes (29–32). Alongside, some of these lungworm species parasitize domestic/feral cats (*Felis catus*) and wild cats (*Felis silvestris*) (33–42). Nonetheless, very little is currently known on these infections in free-ranging Eurasian lynxes. The crenosomatid lungworm species *T. brevior* and *C. vismani* parasitize the bronchi and bronchioles of feline definite hosts whilst *A. abstrusus* resides in subpleural parenchyma and alveoli (32, 37, 43, 44). Conversely, the angiotropic nematode *A. chabaudi* parasitizes the pulmonary arteries and the right heart of mainly wild cats (30, 33, 45). Embryonated metastrongyloid eggs are deposited, first-stage larvae (L1) hatch and migrate via lung tissues to larynx/pharynx, are swallowed and shed via defecation into the environment during patency. Thereafter, exogenous L1 infect terrestrial gastropods (i.e., slugs, semi-slugs, snails) acting as obligate intermediate hosts. In gastropods, L1 develop into second-(L2) and infective third-(L3) larval stages within approximately 2–4 weeks, depending on the parasite species. Eurasian lynxes become infected either after ingesting L3-infected gastropods or via consumption of paratenic hosts (amphibians, reptiles, birds, rodents) carrying infective L3. Alternatively, but more unlikely, wild Eurasian lynxes might become infected from standing waters containing dead intermediate hosts and/or released infective L3 (46). In case of *T. brevior*, also lactogenic transmission was recently demonstrated for domestic cats in Italy (42).

Feline troglostrongylosis has gained scientific interest in Europe where it is considered as a spill-over event from wild cats (*F. silvestris*) to feral cats (30, 32, 37, 42, 47–52). Pathological alterations of troglostrongylosis in lynx include multifocal, consolidated, firm tan to gray areas in various lung lobes with thickened alveoli walls, filled with necrotic debris, leukocyte infiltration and degenerated inflammatory cells, as well as parasite larvae and eggs and a lung oedema (32), thereby corroborating histopathological findings of *T. brevior*-infected wild cats (*F. silvestris*) (35, 36, 53). In line, proteinaceous lung oedema was described in wild bobcats (*Lynx rufus*) infected with closely related *Troglostrongylus wilsoni* (54). Of note, a recent study identified *T. brevior* in terrestrial gastropods in South America thereby expanding its geographic distribution (55). Obviously, spill-over of metastrongyloid infections from feral cats to free-ranging Eurasian lynxes or wild cats may occur when sharing the same biome (31). As already stated, only few studies exist on patent metastrongyloid infections in wild Eurasian lynxes and on their impact on population health (29, 31, 32, 37, 38). Therefore, the current study aims to add epizootiological data by evaluating not only patent metastrongyloid infections in free-living Eurasian lynxes but also in gastropod intermediate hosts in the Harz Mountains, being habitat of the largest *L. lynx* population in Germany.

## 2 Material and methods

### 2.1 Study area

**Figure 1:**
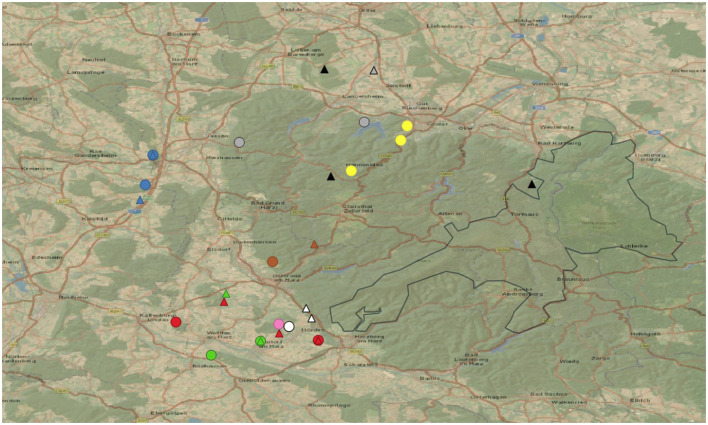
Study area with fecal sampling sites (dots) and gastropod collection sites (triangles) according to the associated lynxes (for clarification which lynx is associated see Table 1). The Harz National Park (black outline drawing), and country roads (orange) in the area of study.

Collection sites were allocated along the western part of the Harz Mountains nearby the Harz National Park (HNP; 51.6946953, 10.5674415) in Germany. This mountainous area outside the HNP is characterized by opened landscapes, vast forested and meadow areas, provincial towns and some large country roads. An illustration of the study area is given in Figure 1, the respective geographic map was generated by QGIS V.3.28.1 (QGIS Geographic Information System. QGIS Association. http://www.qgis.org).

### 2.2 Collection of scat samples and gastropods at Eurasian lynx killing sites

**Figure 2:**
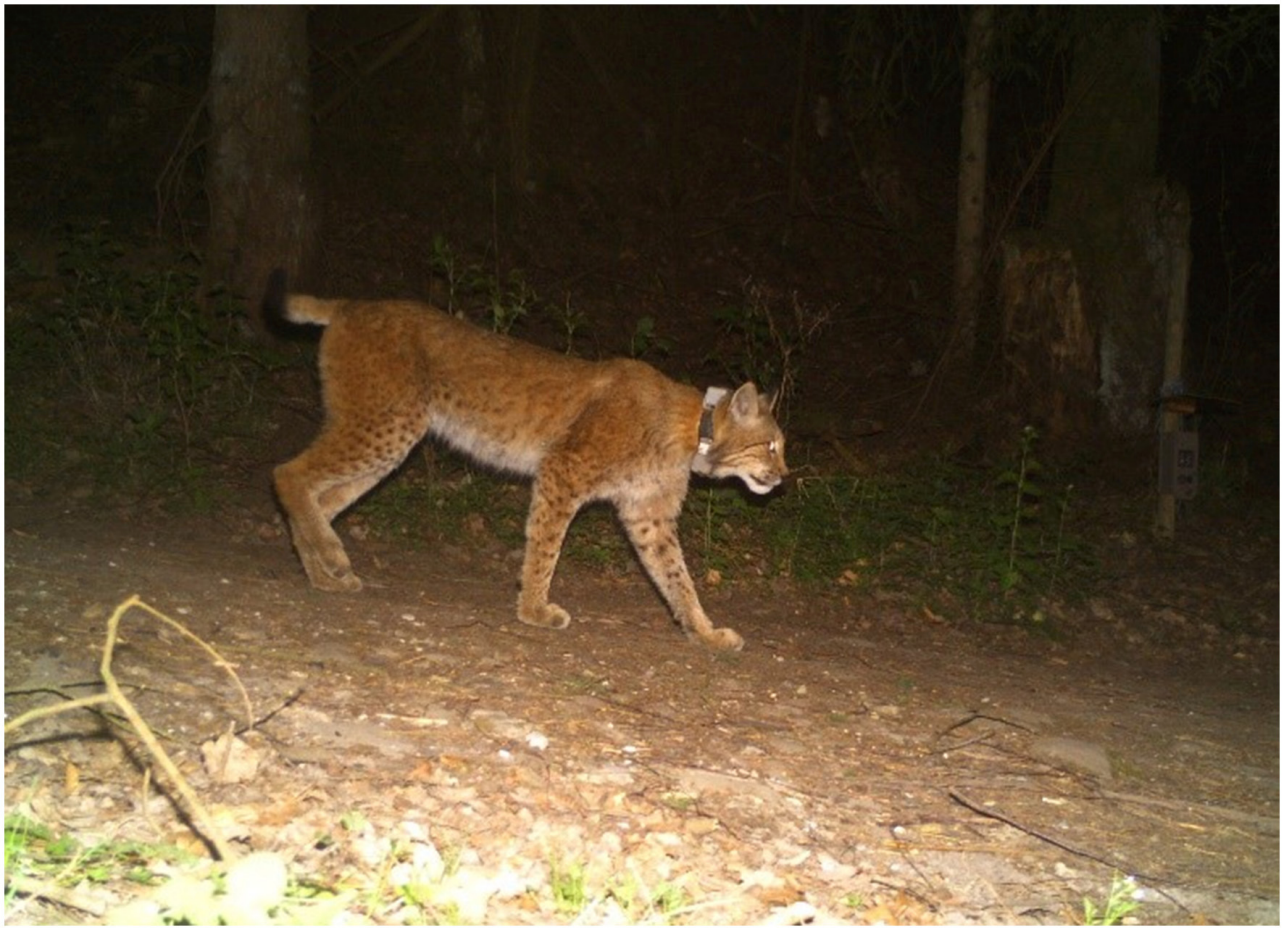
A wild Eurasian lynx (*Lynx lynx*) wearing a GPS collar.

**Figure 3:**
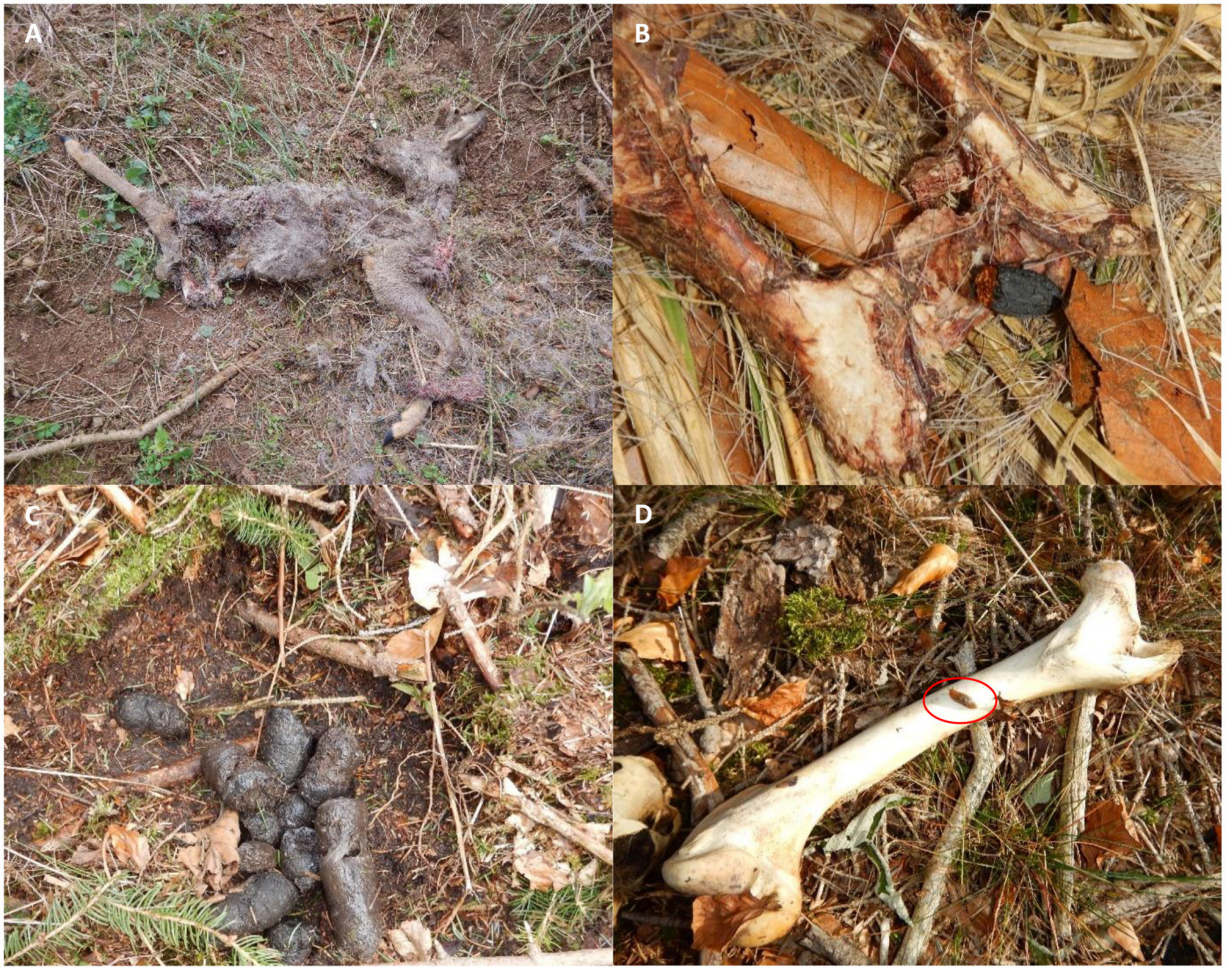
A typical Eurasian lynx (*Lynx lynx*) killing site at the Harz Mountains. **(A)** Leftovers of a killed roe deer (*Capreolus capreolus*). Initially the carcass was found entirely covered with grass, leaves, branches and soil particles; **(B)** a red-breasted carrion beetle (*Oiceoptoma thoracicum*) feeding on meat leftovers of a roe deer pelvis. **(C)** An excavated Eurasian lynx faecal sample which was also found entirely hidden under grass, leaves and branches. **(D)** Terrestrial slug (*Arion* sp.; indicated by red circle) found on a bone.

Wild Eurasian lynxes sampled in this study included 6 young/sub-adult animals and an adult male. Four of these lynxes (F10, M18, M19, and M20) were rehabilitated animals, which were originally found as orphans and raised in enclosures before being released again into the wild. The other three animals (F11, F12, and M22) were captured weak, and were rehabilitated before release, none of them showed any clinical respiratory signs. All animals were equipped with GPS/GSM-transmitting collars (VECTRONIC AEROSPACE, Berlin) by the staff of the HNP before release (see Figure 2). Moreover, parasitological examinations of collared juvenile Eurasian lynxes were performed, which excludes M22 which was captured as an adult animal. All juvenile animals were tested with copromicroscopy and were all found positive for *Toxocara cati* and thus received anthelmintic treatments (Ivomec^®^, ivermectin, 0.5 mg/kg, Boehringer Ingelheim), before being released again. Lynx killing sites were identified using two handheld GPS devices (GPSMAP^®^ 64s and GPSMAP^®^ 65s, Garmin, Olathe, USA) and by profiting from received repeated collar-transmitted signals from distinct geographic spots, thereby proving the presence of lynxes at this locations (see above). In the current study, hidden prey animals included roe deer (*Capreolus capreolus*), hares (*Lepus europaeus)* and red foxes (*Vulpes vulpes*). A variety of arthropods (e.g., flies, maggots, carrion beetles, ants and isopods) as well as different terrestrial gastropods (i.e., slugs, semi-slugs and snails) were found in close proximity to killing sites (please refer to Figures 3, 5). In total, 41 killing sites or Eurasian lynx habitats were identified via GPS tracking and on 25 sites, scat- and/or gastropod samples were successfully collected.

**Figure 4:**
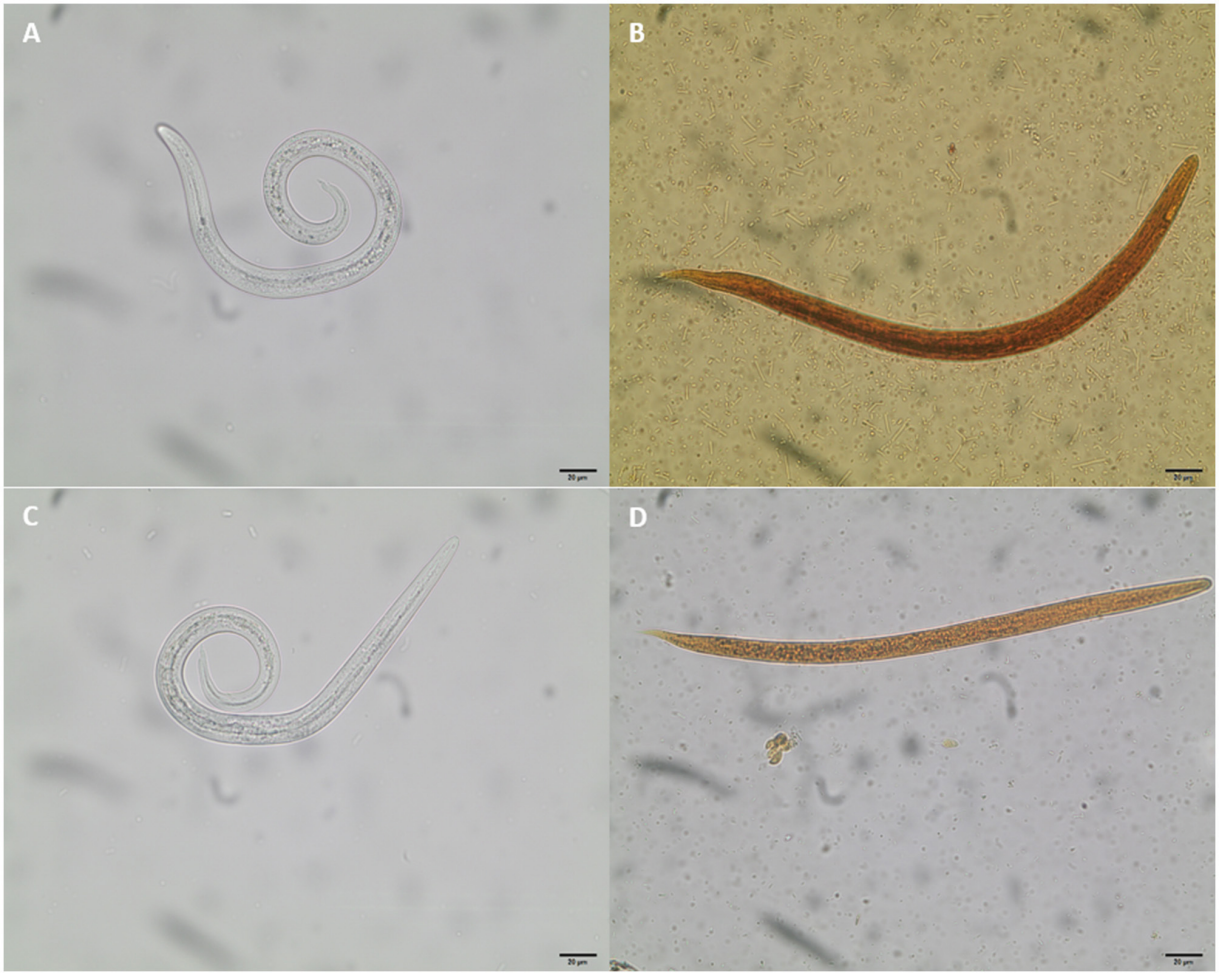
Coproscopic findings of metastrongyloid first-stage larvae (L1) in Eurasian lynx fecal samples. **(A)** *Aelurostrongylus abstrusus*, 366 μm length, 15 μm width; **(B)** *Troglostrongylus brevior*, 340 μm length, 19 μm width; **(C)** *Angiostrongylus* sp.-larvae 370 μm length, 15 μm width; **(D)** *Crenosoma* sp. 314 μm length, 14 μm width.

**Figure 5:**
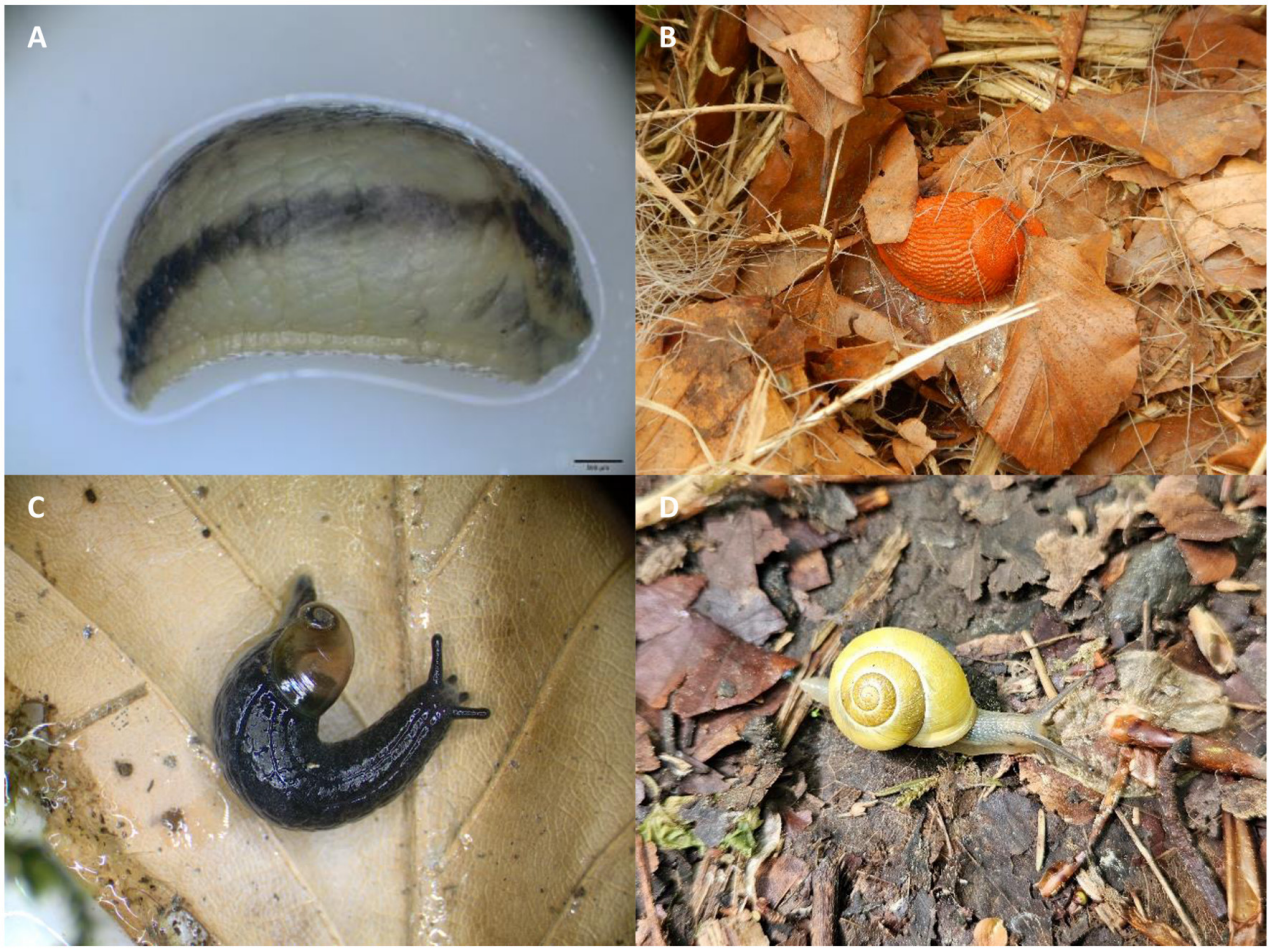
Example of collected terrestrial gastropods. **(A)** Juvenile *Arion sp*. (breathing hole in cranial part of the mantle); **(B)** adult *Arion* cf. *rufus* (breathing hole in cranial part of the mantle, rocking behavior, orange coloration); **(C)** *Daudebardia* cf. *brevipes* (grey-blueish body, specific shell formation); **(D)** *Cepaea hortensis* (yellow mouthlip of the shell).

Overall, 24 individual fecal samples originating from the seven individuals mentioned above, were collected during six visits of killing sites between March 2022 and February 2023. As mentioned before these samples could be associated with the different individual lynx by the sampling methods here used. Following this 3/24 samples originated from M18 (12,5%), 6/24 from M19 (25%), 1/24 from M20 (4,2%), 3/24 from M22 (12,5%), 3/24 from F10 (12,5%), 4/24 from F11 (16,6%), 1/24 from F12 (4,2%) and 3/24 from a meeting point of F12 + M22 (12,5%). Scat samples were identified based on characteristic morphology, size, composition (mainly containing roe deer hair), emission of lynx-specific odor, as well as the feline specific covering behavior of the feces with surrounding material. All the before mentioned characteristics in combination lead to the diagnosis of lynx feces. Collected scat samples were labeled, kept at 4°C and immediately transferred to the Institute of Parasitology of the Justus Liebig University Giessen for further parasitological analysis. In cases of uncertainty of the scat origin, fecal samples were additionally analyzed by molecular approaches at the Centre for Wildlife Genetics, Senckenberg Research Institute and Natural History Museum, Gelnhausen, Germany (for detailed description of this methodology refer to Section 2.3).

Besides scat samples, a total of 153 terrestrial gastropods were collected during 2021 and 2023 at 14 previously selected GPS-tracked killing sites or in Eurasian lynx habitats during scat sampling (please refer to Table 1, Figures 1, 5), and surrounding areas (up to 1 km radius). All gastropods were collected manually by wearing gloves in search for humid mollusc hiding places (i.e., beneath leaves, rocks or rotten wood) or in close proximity to killing sites. Some slugs were directly collected from carcasses, bones or beneath carcasses, as illustrated in Figure 3. All GPS-identified lynx killing sites were visited during daytime to avoid disturbance of nocturnal Eurasian lynxes and prey animals and with a time delay of at least 3 days to not disturb lynxes in their feeding behavior.

**Table 1:** Sample collection sides with associated lynxes.

**Sampling side associated lynx**	**Collection date**	**Collection side**	**Fecal sample**	**Gastropod species (number)**	**Morphological results (number of samples) in lynx fecal sample = (L); in gastropod sample = (G)**	**Molecular results (number of samples) in lynx fecal sample = (L); in gastropod sample = (G)**	**Prey species and other information on sampling side**
**M18** 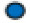 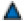							
	4/8/2022	51.83913°, 10.10655°	Yes (1x)	/	/	/	Roe deer
	4/9/2022	51.87350°, 10.11538°	Yes (2x)	Unidentified gastropod (1)	/	/	Roe deer
	4/24/2022	51.82179°, 10.10059°	/	*Arion* sp. (3), *Arion vulgaris* (1)	/	/	Roe deer
**M19** 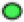 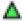							
	4/10/2022	51.71321°, 10.20021°	/	*Cepaea sp*. (6), *Discus rotundatus* (4), *Limax maximus* (1)	/	*/*	/
	4/23/2022	51.65810°, 10.24001°	Yes (1x)	*Cepaea sp*. (3), *Arion* sp. (1), *Deroceras reticulatum* (1), *Limax cinereoniger* (1), *Limax maximus* (1)	*Angiostrongylus* sp. (1) (L)	*/*	3 red foxes
	2/13/2023	51.64184°, 10.18289°	Yes (5x)	/	/	*/*	/
**M20** 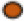 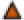							
	3/17/2022	51.770390°, 10.302170°	/	*Arion* sp. (3)	/	/	
	3/21/2022	51.75012°, 10.253870°	Yes (1x)	/	*A. abstrusus* (1) (L), *Angiostrongylus* sp. (1) (L)	*A. abstrusus* (1)(L)	
**M22** 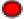 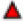							
	3/27/2022	51.68020°, 10.14202°	Yes (2x)	*/*	*Crenosoma* sp. (L) (1), *T. brevior* (L) (1)	/	Roe deer
	4/10/2022	51.667140°, 10.261500°	/	*Arion sp. (3), Cepaea sp. (2), Discus rotundatus (1), Limax cinereoniger (1)*	/	/	Roe deer
	4/21/2022	51.70369°, 10.19776°	/	*Arion* sp. (2), *Arion* cf. *rufus* (1)	/	/	Roe deer; *Arion* cf. *rufus* see Figure 5
	4/24/2022	51.65908°, 10.30684°	Yes (1x)	*Limax cinereoniger* (2)	*T. brevior* (1) (L)	/	Bird
**F10** 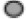 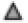							
	3/27/2022	51.91183°, 10.35997°	Yes (1x)	/	*T. brevior* (1) (L)	*T. brevior* (1) (L)	Hare
	4/8/2022	51.88828°, 10.21545°	Yes (2x)	/	*T. brevior* (L) (2)	/	Roe deer + resting place
	4/22/2022	51.97175°, 10.37115°	/	*Cepaea* sp. (1)	/	/	Roe deer
**F11** 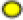 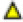							
	4/11/2022	51.85538°, 10.34501°	Yes (1x)	/	/	/	Roe deer
	1/15/2023	51.89079°, 10.40236°	Yes (2x)	/	/	/	Roe deer
	1/15/2023	51.90740°, 10.40969°	Yes (1x)	/	/	/	Roe deer
**F12** 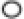 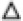							
	4/11/2022	51.68467°, 10.29901°	/	*Arion* sp. (6), *Cepaea* sp. (2), *Daudebardia* sp. (2), *Aegopinella* sp. (1), *Daudebarida brevipes* (1), *Daudebardia rufa* (1)	/	/	Hare; for *Daudebardia* sp. see Figure 5
	4/11/2022	51.69615°, 10.29277	/	*Arion* sp. (6), *Limax cinereoniger* (1)	/	/	Hare
	1/17/2023	51.67504°, 10.27293°	Yes (1x)	/	/	/	Roe deer
**F12**+**M22** 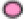							
	3/20/2022	51.67781°, 10.261090°	Yes (3x)	/	*A. abstrusus* (L) (1)	*T. brevior* (L) (1)	Mating point F12 + M22; molecular result were found in positive fecal sample (L)
**No associated lynx** 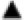							
	6/15/2021	51.97298°, 10.31394°	/	*Arion* sp. (30), *Helicodonta obvoluta* (4), *Limax maximus* (3), *Limax cinereoniger* (2), unidentified gastropod (2), *Cochlodina laminata* (1), *Discus rotundatus* (1)	*A. vasorum* (2) (G)	/	Langelsheim
	6/16/2021	51.83997°, 10.55407°	/	*Arion* sp. (32), Cepaea sp. (3), *Limax maximus* (3), *Discus rotundatus* (2), *Succinea putris* (2), *Cochlodina laminata* (1), *Limax cinereoniger* (1), unidentified gastropod (1)	/	/	Bad Harzburg
	4/11/2022	51.848972°, 10.321411°	/	*Arion* sp. (3)	/	/	Roe deer
	2022	/	/	*Limax maximus* (3)	/	/	Found in the Harz region but with no exact location

### 2.3 Molecular analysis of lynx feces

At the Centre for Wildlife Genetics (Senckenberg Research Institute and Natural History Museum, Gelnhausen, Germany) DNA from fecal samples were extracted, this and the following steps were performed by using the QIAamp Fast DNA Stool Mini Kit (Qiagen, Germany) and using the Qiacube-Robotic-System (Qiagen, Germany). Two mitochondrial markers were employed for species identification. The first marker consisted of the two primers L15995 (5′-CTCCACTATCAGCACCCAAAG-3′) and H16498 (5′-CCTGAAGTAAGAACCAGATG-3′) (56) and was used for general confirmation of a mammal species. The second marker consisted of the two primers LF4 (5′-GACATAATAGTGCTTAATCGTGC-3′) (57) and H16498 (5′-CCTGAAGTAAGAACCAGATG-3′) (58) detecting particularly members of the family Felidae. For PCR, 5 μl SensiFAST SYBR No-ROX Kit (Biocat, Germany), 0.4 μl of the respective primer pair, 1.2 μl of nucleic acid-free Water (Carl Roth, Germany) and 3 μl of DNA extract were mixed. The cycling protocol included 1 × 3 min at 95°C, 40 × 95°C for 5 s and 60°C for 30 s, 10°C for storage. Before Sanger sequencing, PCR products were purified by ExoSAP-IT (Thermofisher Scientific, USA) following the manufacturer's instructions. The BLAST tool (59) was used for species determination of the obtained sequences.

### 2.4 Detection of metastrongyloid first-stage larvae in fecal samples

Fecal samples were processed at the day of collection by using the standard Baermann funnel technique (60, 61). After 24 h of incubation, samples were microscopically analyzed using an Olympus BH-2^®^light microscope (Olympus, Tokyo, Japan) equipped with a digital camera (SC30^®^, Olympus, Tokyo, Japan). Metastrongyloid L1 were morphologically characterized, and in cases of high larval motility, treated with Lugol's iodine solution [iodine-potassium iodide solution according to Lugol (1% iodine), Carl Roth, Germany] to immobilize larvae (62). Lungworm larvae were identified to genus level according to their typical morphological and morphometric characteristics using several larvae per sample [i.e., body length, detail of anterior extremity, oesophagal shape (non-rhabditiform) and length (1/3–1/2 the length of larvae), and typical tail morphology] (31, 32, 34, 35, 47, 48, 63, 64) (see Figure 4).

### 2.5 Gastropod digestion for metastrongyloid larvae detection

Gastropods were first identified to genus level via morphological characteristics, then cryo-euthanized and stored at −20°C until further processing (55), artificially digested and sieved according to Penagos-Tabares et al. (55). In brief, frozen gastropods were cut into small pieces and immersed in a digestion solution [10 g pepsin powder 2000 FIP-U/g (Carl Roth, Germany), 8.5 g NaCl, 30 mL HCl 37% (Carl Roth, Germany) adjusted to 1 L by distilled water] for 3 h at 37°C in 50 ml sterile plastic tubes (Greiner) under permanent shaking. After digestion, samples were sieved through a 300 μm pore-sized metal sieve (Retsch) to remove undigested material/debris and then passed through a 25 μm pore-sized metal sieve (Retsch). Remnants of the latter sieving process, were then transferred to 10 ml tubes and sedimented at 800 *g* for 5 min at room temperature (RT). The sediments were examined microscopically for the presence of metastrongyloid larvae (Olympus BH-2^®^, Olympus, Tokyo, Japan).

### 2.6 Molecular identification of metastrongyloid species

All larvae-positive samples (*n* = 11, 9 fecal- and 2 gastropod samples) were additionally analyzed by molecular techniques. Therefore, all larvae from each Baermann sediment were collected via careful pipetting and each sediment were analyzed individually by metastrongyloid-specific PCRs, and finally sequenced to species level. Therefore, DNA was isolated from larvae using a commercial kit (DNeasy Blood and Tissue Kit^®^, Qiagen, Hilden, Germany). PCRs were performed using the universal nematode primers NC1 (5′-ACGTCTGGTTCAGGGTTGTT-3′) and NC2 (5′-TTAGTTTCTTTTCCTCCGCT-3′) (65). Using a total reaction volume of 50 μl, HOT FIREPol^®^ Blend Master Mix (Solis BioDyne, Tartu, Estonia) and 5 μl of DNA template, cycling was performed at the following conditions: denaturation at 95°C for 15 min, 35 cycles of denaturation at 95°C for 20 s, annealing at 52°C for 30 s and extension at 72°C for 30 s, followed by a final elongation step at 72°C for 5 min as reported elsewhere (65). In cases when metastrongyloid PCRs yielded negative or inconclusive results, and the quantity of amplicon-DNA from initial PCR was low, a second nested conventional PCR was conducted using the primers NC1 (5′-ACGTCTGGTTCAGGGTTGTT-3′) and MetR (5′-CCGCTAAATGATATGCTTA-3′) (66). Obtained amplicons were purified via gel electrophoresis, sent to a commercial sequencing service (LGC Genomics, Berlin, Germany) and analyzed by BLAST search (http://www.ncbi.nlm.nih.gov/BLAST/; accessed on 15 December 2022).

## 3 Results

### 3.1 Locations of GPS-tracked killing sites

GPS-tracked lynx killing sites included areas of wastewater treatment plants, abandoned quarries, private shooting grounds and former ammunition depots, all in vicinity to wooded areas (see Figure 1).

### 3.2 Occurrence of metastrongyloid infections in wild Eurasian lynxes

Out of 24 lynx fecal samples, 37.5% (9/24) revealed positive for metastrongyloid L1 and 57.1% (4/7) Eurasian lynxes showed metastrongyloid-positive fecal samples for at least one lungworm species (for details see Table 1). All L1 were identified as parasitic nematode larvae belonging to the family Metastrongylidae. In total, four different cardiopulmonary parasites were identified to genus level: *Aelurostrongylus, Angiostrongylus, Crenosoma*, and *Troglostrongylus* (see Figure 4).

Three PCR products obtained from Eurasian lynx fecal samples proved positive for feline metastrongyloid-specific DNA and were thereafter analyzed by sequencing. Based on molecular analyses, two parasitic larvae were additionally identified to species level as *T. brevior* and *A. abstrusus*. However, the molecular identification of *Angiostrongylus* and *Crenosoma* L1 remained un-conclusive. The gene sequencing results were deposited at GenBank^®^ under the accession numbers: OQ225253 (for *A. abstrusus*) and OQ222066 + OQ222065 (for *T. brevior*), respectively. In one Eurasian lynx (M20), a patent co-infection with *A. abstrusus* and *Angiostrongylus* sp. was detected. One lynx (M19) showed in one sample *Angiostrongylus* sp. L1 whereas in a later scat sample this finding could not be reconfirmed. Another lynx (M22) showed *Crenosoma* sp. L1 and *T. brevior* L1 in two different samples from the same location, in a later sample *T. brevior* L1 could be reconfirmed.

### 3.3 Gastropod species diversity and metastrongyloid infections in gastropods

The most common gastropod species found at lynx killing sites or in the surroundings were slugs of the genus *Arion* (59.5%; 91/153), followed by snails of the genus *Cepaea* (11.1%; 17/153) and the Leopard slug (*Limax maximus*, 10.5%; 16/153). Rare semi-slug species of the genus *Daudebarida* (2.6%; 4/153) with small translucent shells were also collected (see Figure 5). For more details on terrestrial gastropod species diversity please refer to Table 1.

**Figure 6:**
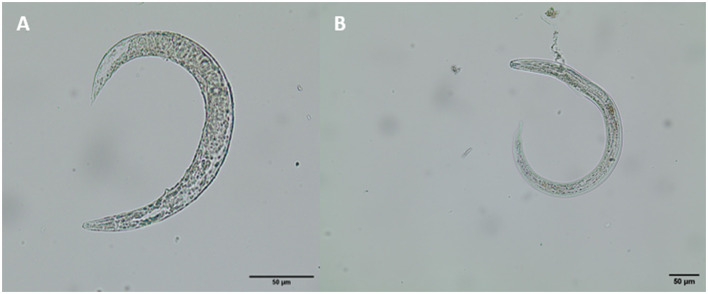
Lungworm larvae found in *Arion* cf. *vulgaris* **(A)** *A. vasorum* L1, **(B)** *A. vasorum* L3.

Of digested terrestrial gastropods (*n* = 153), 1.3% of them (2/153) contained metastrongyloid larvae and were identified as *A. vasorum* (see Figure 6). All two positive slugs belonged to the genus *Arion*. Referring to slug larval burden, one *Arion* slug carried two *A. vasorum* larvae whilst the other one proved highly infected with a total larval burden of 34 larvae. Overall, all three larval development stages of *A. vasorum*, i.e., L1, L2, and L3, were found in the latter slug.

## 4 Discussion

The killing site-based, non-invasive sample collection included several advantages like an efficient, un-molested scat and gastropod collection leading to less harm and stress for both humans and animals and being in accordance to current animal welfare and wildlife conservation strategies in contrast to other sampling methods, which might require stressful animal capture. Thus, GPS-based identification of killing sites (see above) seems feasible for non-invasive scat sample- and terrestrial gastropod collections.

Based on the biological behavior of lynxes, actual killing sites were sometimes hard to discover in the field, since lynxes typically deeply hide their prey under thorny bushes, foliage and branches or deposit them in very remote areas. In most cases, feces were found entirely covered by leaves and branch piles reflecting typical feline behavior. Most of the places with killing sides share the characteristic of beeing human-dominated areas, with a low level of human interaction.

The current study is based on a lynx population consisting of sub-adult and adult individuals, which were re-introduced into nature after their capture and rearing (below 1 year). Proceedings of re-introduction included obligatory anthelminthic treatments of sub-adult lynxes (M18, M19, M20, F10, F11, and F12) with ivermectin to eliminate *T. cati* and potential other nematode infections before release. It is to mention that currently there is no explicit study which shows the specific effectiveness of ivermectin against all the metastrongyloid lungworms, which were found in this survey. However there are different reports which show the effectiveness of ivermectin against lungworms of the genus *Crenosoma* (67, 68). Against *Aelurostrongylus abstrusus* ivermectin seems to have an incomplete effectiveness (69, 70). For the use of ivermectin against *Angiostrongylus* sp. and *Troglostrongylus* sp. in felids there is currently insufficient scientific knowledge. We therefore recommend to review reintroduction protocols for wild felids using ivermectin and maybe switch to better working compounds if the aim is to eradicate metastrongyloid lungworm infections. If ivermectin is to be used, the animals should be tested again with the Baermann funnel after treatment to detect possibly surviving metastrongyloid lungworms and if the result is positive, the animals should be treated with another more specific compound. A complete absence of metastrongyloid lungworm infections in the reintroduced lynxes, mentioned in this manuscript, prior their release into the wild, can therefore not to be ruled out and given that the results of the copromicroscopic analysis yielded negative for metastrongyloid lungworm infections, it seems still highly feasible to assume that patent metastrongyloid infections detected in the current study were acquired within the study area either by ingesting metastrongyloid-infected gastropod intermediate hosts and/or after consumption of infected paratenic hosts including amphibians, reptiles, birds and rodents.

The metastrongyloid genera *Aelurostrongylus, Angiostrongylus*, and *Troglostrongylus* have previously been described not only in wild Eurasian lynxes but also in wild cats (*F. silvestris*) and domestic cats (30, 37, 50). In contrast, *Crenosoma* infections in wild felids were reported exclusively for *L. lynx* and identified as *C. vismani* (29). Of note, current parasitological results include the broadest metastrongyloid species diversity for wild Eurasian lynxes compared to former reports (31, 37, 38, 71). This species diversity might be explained by the current method, i.e., examination of fresh feces by the Baermann funnel, which allows the detection of living larvae. Of note, this technique is still considered as gold standard for lungworm larvae diagnostics (72–80). In contrast, other studies on Eurasian lynx lungworms either analyzed preserved (i.e., fixed or frozen) fecal samples or examined carcasses and/or did not apply the Baermann funnel technique, thereby potentially reducing the sensitivity of lungworm detection (31, 38, 71, 81). Another explanation for current parasite diversity may be related to the age structure of analyzed Eurasian lynxes, consisting mainly of sub-adult animals. Correspondingly, juvenile domestic cats and young wild cats also showed a broader range of metastrongyloid species, thereby being predisposed for these cardiopulmonary infections by age, we would assume that this will be the case for Eurasian lynx as well (30, 48, 51, 82–84).

A recent study on endoparasites of free-ranging Eurasian lynxes (*n* = 24) of the Harz Mountains reported a prevalence of 12.5% (3/24) for metastrongyloid L1, among them *Angiostrongylus* spp.-like larvae and *A. abstrusus*, nonetheless neither *T. brevior-* nor *Crenosoma* spp.-larvae were detected in this study (31). Interestingly, the present study revealed a lungworm species that has never been reported before in free-ranging German Eurasian lynxes, the metastrongyloid lungworm *Troglostrongylus brevior*. Moreover, current findings on troglostrongylosis represents the third-ever report in literature for this feline host species in Europe. Hence, the first and second report on *T. brevior* infections in Eurasian lynxes came from Bosnia and Herzegovina in 2015 (32) and from Romania in 2022 (37), respectively. Conversely, in North America the closely related species *T. wilsoni* was reported to occur in Canadian lynxes (*Lynx canadensis*) (85) and in wild bobcats (*Lynx rufus*) (54, 86–88). In line with rare reports on lynx troglostrongylosis, the occurrence of aelurostrongylosis in Eurasian lynxes has only been described for three countries so far, i.e., Switzerland, Poland and Germany (31, 38, 89). *Troglostrongylus* seems to be of more clinical concern, than *A. abstrusus* as they seem to show in general a more severe clinical picture (90–92). In Switzerland, analyses of 58 fecal samples from dead *L. lynx* were in five cases (9%) positive for *A. abstrusus*. Interestingly, in one of the examined Swiss Eurasian lynx, histopathological findings unveiled a multifocal mild granulomatous pneumonia (89). In Poland, a much higher *A. abstrusus* prevalence of 21% was reported (38). Current findings re-confirm *A. abstrusus* as circulating in free-ranging Eurasian lynxes in Germany and highlight the importance of regular monitoring on gastropod-borne aelurostrongylosis (31). Recently, feline crenosomosis was reported in wild Eurasian lynxes in Latvia. Based on morphological and morphometric characteristics, *C. vismani* was identified as the related infective pathogen (29). In the current study, we failed to identify the *Crenosoma* species for the detected larvae by molecular tools. It has to be noted that, it cannot be ruled out, that the here identified *Crenosoma*-L1 might also have originated from an infected prey animal [i.e., red fox (*Crenosoma vulpis*) or European hedgehog (*Crenosoma striatum*)] (41, 93–99). Accordingly, three carcasses of killed red foxes were found at one killing site. Equally, current findings on *Angiostrongylus-*L1 might have originated from other infected prey, predator/mesopredator living within the same biome. Hence, interactions of wild Eurasian lynxes with badgers (*Meles meles*), racoons (*Procyon lotor*) and wild cats sharing the same habitats of the Harz Mountains might result in spill-overs and spill-backs of *Angiostrongylus* spp.-infections as previously postulated (27, 30, 31, 100, 101). Particularly feral cats and domestic dogs may pose a risk in reverse of spill-overs for endangered Eurasian lynxes (31). Wildlife studies have already demonstrated that especially feral cats play a significant role in transmitting parasites to wild lynx populations as reported for re-introduced Iberian lynxes in Spain and Portugal (27, 100, 101).

In the present study, we failed to molecularly confirm the endemic *A. chabaudi* parasite to species level for the current German lynx samples. Nevertheless, this species has already been reported for wild cats in Germany (30), and from other European countries (35, 36, 102). However, patent *A. chabaudi*-infections have not yet been detected in wild Eurasian lynxes. Given that wild cats and Eurasian lynxes cohabit the same biome in the Harz Mountains, transmission of *A. chabaudi* seems plausible through consumption of either infected intermediate hosts or paratenic hosts (31, 64, 103), but further investigations are needed for conclusive data on lynx angiostrongylosis in Germany.

In this study lungworm larvae only of the species *A. vasourm* were found in gastropods of the genus *Arion* sp. The origin of infection of the sampled lynxes, with the lungworms, which were found during the analysis of the Baermann funnel, has to be investigated in further studies. Until this time their way of infection is speculative. Felids tend to get infected with metastrongyloid lungworms by ingesting paratenic hosts (e.g., rodents and birds) rather than by ingesting gastropods, this case seems also feasible for Eurasian lynxes and is therefore a highly reasonable cause of infection (39, 104). Wild cats (*F. silvestris*) seem to be a reservoir for *T. brevior* (49), and as they are occurring syntop with the Eurasian lynx in the Harz Mountains they could be a source of infection. The majority of the collected slugs belonged to the species *Arion* cf. *vulgaris*, which is in accordance to other studies the most common slug species in Germany and mainly found in urbanized areas due to its synanthropic behavior (105). Nonetheless, also *Arion* cf. *rufus* slugs were here found in the lynx biomes of the Harz Mountains. Unfortunately, the species determination of collected *Cepaea* snails was impossible since pivotal morphological characteristics for proper identification (e.g., color of the mouthlip of the shell, internal morphology of reproductive organs) were missing due to beginning autolysis or fragmentation of the shell (106). The susceptibility of gastropod species to lungworm infections is explained by different factors e.g., intermediate host behavior and size of the gastropod (52, 107, 108). The exact parameters which influence the susceptibility of gastropod species have to be investigated in further studies, for example with a broader sample size or experimental infections. The prevalence of metastrongyloid lungworms in gastropod intermediate hosts has already been done in several studies (52, 55, 109).

Considering present metastrongyloid findings in digested gastropod intermediate hosts, exclusively *A. vasorum* larvae in two *Arion* cf. *vulgaris* slugs were found. Most probably these infections result from marked coprophagic behavior of this slug species (16, 110) when compared to other investigated gastropod genera (e.g., *Cepaea* and *Daudebardia*), what makes them good intermediate hosts. When referring to definite hosts of *A. vasorum*, the most predominant species in the Harz Mountains are red foxes followed by gray wolves (*Canis lupus*), besides domestic dogs, while felines are usually considered as rare and inadequate hosts (111, 112). Especially red foxes are well-known to show high *A. vasorum* prevalences in Germany (93, 113). Moreover, it cannot be ruled out that the presence of *A. vasorum* larvae in *Arion* slugs was due to intermediesis, where nematode stages are transferred from one infected gastropod to another by carnivorous behavior or by contact with L3 released from dead intermediate hosts (114, 115).

## 5 Conclusion

To the best of current knowledge, this work represents the first report on patent *T. brevior-*infections in wild Eurasian lynxes in Germany. Additionally, current data re-confirmed patent *A. abstrusus* infections to be circulating in the *L. lynx* population inhabiting the Harz Mountains, which is the largest one in Germany. Our findings emphasize the necessity for additional research on neglected cardiopulmonary diseases, such as feline angiostrongylosis and crenosomosis, in wild Eurasian lynxes to increase the current understanding on their epizootiology, pathogenesis, immunity, and clinical relevance. Moreover, the presence of *A. vasorum* larvae in gastropods was here reported for the first time for the Harz Mountains within the Federal State of Lower Saxony. Even though our study is limited in terms of low animals numbers, its significance lies in the importance of exploring wildlife-associated parasitoses in a non-disturbing manner to uncover potential threats to endangered large felid apex predators. Considering the limited knowledge on the pathological and clinical findings induced by lungworm infections in wild felids, regular veterinary monitoring is crucial to evaluate the population health status, which could additionally be done by the examination of samples from other regions as wells as clinical assessments of animals in rescue centers or necropsies of dead animals to get broader insights in metastrongyloid lungworm infections in Eurasian lynxes. These regular veterinary monitoring programs will play a crucial role for future re-introduction programs and should encompass not only the target but also sympatric species.

## Data Availability

The datasets presented in this study can be found in online repositories. The names of the repository/repositories and accession number(s) can be found in the article/supplementary material.

## References

[1] Sergio F Newton I Marchesi L. Top predators and biodiversity. Nature. (2005) 436:192–192. 10.1038/436192a 16015318

[2] Ripple WJ Estes JA Beschta RL Wilmers CC Ritchie EG Hebblewhite M. Status and ecological effects of the world's largest carnivores. Science. (2014) 343:1241484. 10.1126/science.1241484 24408439

[3] Crooks KR Soulé ME. Mesopredator release and avifaunal extinctions in a fragmented system. Nature. (1999) 400:563–6. 10.1038/23028

[4] Soulé ME Bolger DT Alberts AC Wrights J Sorice M Hill S. Reconstructed dynamics of rapid extinctions of chaparral-requiring birds in urban habitat islands. Conser Biol. (1988) 2:75–92. 10.1111/j.1523-1739.1988.tb00337.x

[5] Laundré JW Hernández L Altendorf KB. Wolves, elk, and bison: reestablishing the ‘landscape of fear' in Yellowstone National Park, U.S.A. Can J Zool. (2001) 79:1401–9. 10.1139/z01-094

[6] Burgos T Fedriani JM Escribano-Ávila G Seoane J Hernández-Hernández J Virgós E. Predation risk can modify the foraging behaviour of frugivorous carnivores: Implications of rewilding apex predators for plant–animal mutualisms. J Anim Ecol. (2022) 91:1024–35. 10.1111/1365-2656.13682 35322415 PMC9311824

[7] Sarasola JH Zanón-Martínez JI Costán AS Ripple WJ. Hypercarnivorous apex predator could provide ecosystem services by dispersing seeds. Sci Rep. (2016) 6:19647. 10.1038/srep19647 26791932 PMC4726145

[8] Krawczynski R. 6.5 Kadaver. In: Naturnahe Beweidung und NATURA 2000 - Ganzjahresbeweidung im Management von Lebensraumtypen und Arten im europäischen Schutzgebietssystem NATURA 2000. 2nd, ed. Bad Sassendorf: Arbeitsgemeinschaft Biologischer Umweltschutz (2019). p. 411.

[9] Krawczynski R Wagner HG. Leben im Tod - Tierkadaver als Schlüsselelemente in Ökosystemen. Naturschutz Landschaftsplanung. (2008) 40:261–4.

[10] Beekers B Gauggel KF Xiaoying G Haas D Krawczynski R Lysakowski B. Mitteleuropäische Wirbeltierarten an Kadavern. Säugetierkundliche Informationen, Jena. (2017) 10:389–406.

[11] Gu X Haelewaters D Krawczynski R Vanpoucke S Wagner HG Wiegleb G. Carcass ecology – more than just beetles. Entomolog Ber. (2014) 74:68–74.

[12] Gu X Wagner HG Krawczynski R. Zur Bedeutung toter Großtiere für die Biodiversität. In: Natürliche Weidelandschaften – eine Versöhnung zwischen Landwirtschaft und Naturschutz (2010).

[13] Krawczynski R. Wirbeltiere an Aas – Erfahrungen aus sechs Jahren Forschung in Brandenburg. In: Neues Leben aus alten Leichen – Aktuelles aus der Aasökologie und der Forensik. Schwedt/Oder (2013).

[14] Schwegmann S Storch I Bhardwaj M. Use of viscera from hunted roe deer by vertebrate scavengers in summer in central European mountainous mixed forest. Wildlife Biol. (2023) 2023:e01117. 10.1002/wlb3.01117

[15] Melis C Selva N Teurlings I Skarpe C Linnell JDC Andersen R. Soil and vegetation nutrient response to bison carcasses in Białowieża Primeval Forest, Poland. Ecol Res. (2007) 22:807–13. 10.1007/s11284-006-0321-4

[16] Kozłowski J. The distribution, biology, population dynamics and harmfulness of *Arion lusitanicus* Mabille, 1868 (gastropoda: pulmonata: arionidae) in Poland. J Plant Protect Res. (2007) 47:119–230.

[17] Nowell K Jackson P. Status survey and conservation action plan wild cats. In: Nature. Switzerland (1996). p. 101–6. Available online at: https://www.nature.com/articles/188716b0 (accessed June 8, 2023).

[18] Kaczensky P Chapron G. Status, management and distribution of large carnivores – bear, lynx, wolf & *wolverine – in Europe*. Part 2 Country Reports (2012).

[19] von Arx M. Lynx lynx (amended version of 2018 assessment). The IUCN Red List of Threatened Species 2020 (2020).

[20] Kaczensky P Chapron G. Status, management and distribution of large carnivores – bear, lynx, wolf & *wolverine – in Europe*. Part 1 General report (2012).

[21] Breitenmoser U Breitenmoser-Würsten C Lanz T von Arx M Antonevic A Bao WD. IUCN Red List of Threatened Species: Lynx lynx. IUCN Red List of Threatened Species (2017). Available online at: https://www.iucnredlist.org/en (accessed June 8, 2023).

[22] Castelló JR Sliwa A Kitchener A. Felids and Hyenas of the World. Princeton: Princeton University Press (2020). Available online at: http://www.jstor.org.ezproxy.uni-giessen.de/stable/j.ctv11hprnk (accessed June 8, 2023). 10.1515/9780691211862

[23] von Arx M Kaczensky P Linnell J Lanz T Breitenmoser-Wörsten C Boitani L. Conservation status of the Eurasian lynx in West and Central Europe. CATnews Special Issue. The Eurasian lynx in Continental Europe(14) (2021).

[24] Reinhardt I Kaczensky P Knauer F. Monitoring von Wolf, Luchs und Bär in Deutschland. Bonn- Bad Godesberg: Bundesamt für Naturschutz (2015). p. 94.

[25] Meinig H Boye P Dähne M Hutterer R Lang J. Rote Liste und Gesamtartenliste der Säugetiere (Mammalia) Deutschlands. Bonn-Bad Godesberg: Bundesamt für Naturschutz (2020). p. 73.

[26] Drouet-Hoguet N Chenesseau D Kunz F Zimmermann F. Situation of the lynx in the Jura Mountains. In: The Eurasian lynx in Continental Europe(14). CATnews Special Issue (2021). p. 29–34.

[27] Figueiredo AM De Carvalho LM González MJP Torres RT Pla S Núñez-Arjona JC. Parasites of the Reintroduced Iberian Lynx (*Lynx pardinus*) and Sympatric Mesocarnivores in Extremadura, Spain. Pathogens. (2021) 10:274. 10.3390/pathogens10030274 33804321 PMC8000845

[28] Ryser-Degiorgis MP Meli ML Breitenmoser-Wörsten C Hofmann-Lehmann R Marti I Pisano SRR. Health surveillance in wild felid conservation: experiences with the Eurasian lynx in Switzerland. In: The Eurasian lynx in Continental Europe(14). CATnews Special Issue (2021).

[29] Stunženas V Binkiene R. Description of *Crenosoma vismani* n. sp., parasitic in the lungs of *Lynx lynx* (L.) (Carnivora: Felidae), with identification key to the species of the genus *Crenosoma* Molin, 1861 (Nematoda: Crenosomatidae). Syst Parasitol. (2021) 98:73–83. 10.1007/s11230-020-09961-1 33184731

[30] Bisterfeld K Raulf MK Waindok P Springer A Lang J Lierz M. Cardio-pulmonary parasites of the European wildcat (*Felis silvestris*) in Germany. Parasit Vectors. (2022) 15:452. 10.1186/s13071-022-05578-z 36471378 PMC9724372

[31] Segeritz L Anders O Middelhoff TL Winterfeld DT Maksimov P Schares G. New insights into gastrointestinal and pulmonary Parasitofauna of wild Eurasian lynx (*Lynx lynx*) in the Harz Mountains of Germany. Pathogens. (2021) 10:1650. 10.3390/pathogens10121650 34959605 PMC8708128

[32] Alić A Traversa D Duscher GG Kadrić M Di Cesare A HodŽić A. *Troglostrongylus brevior* in an Eurasian lynx (*Lynx lynx*) from Bosnia and Herzegovina. Parasites Vectors. (2015) 8:653. 10.1186/s13071-015-1272-9 26692343 PMC4687328

[33] Gherman CM Ionică AM D'Amico G Otranto D Mihalca AD. *Angiostrongylus chabaudi* (Biocca, 1957) in wildcat (*Felis silvestris silvestris*, S) from Romania. Parasitol Res. (2016) 115:2511–7. 10.1007/s00436-016-5032-3 27106235

[34] Gerichter ChB. Studies on the nematodes parasitic in the lungs of Felidae in Palestine. Parasitology. (1949) 39:251–62. 10.1017/S0031182000083827 18112008

[35] Diakou A Psalla D Migli D Di Cesare A Youlatos D Marcer F. First evidence of the European wildcat (*Felis silvestris silvestris*) as definitive host of *Angiostrongylus chabaudi*. Parasitol Res. (2016) 115:1235–44. 10.1007/s00436-015-4860-x 26637312

[36] Stevanović O Diakou A Morelli S Paraš S Trbojević I Nedić D. Severe verminous pneumonia caused by natural mixed infection with *Aelurostrongylus abstrusus* and *Angiostrongylus chabaudi* in a European Wildcat from Western Balkan Area. Acta Parasit. (2019) 64:411–7. 10.2478/s11686-019-00029-9 30756237

[37] Deak G Ionică AM Pop RA Mihalca AD Gherman CM. New insights into the distribution of cardio-pulmonary nematodes in road-killed wild felids from Romania. Parasites Vectors. (2022) 15:153. 10.1186/s13071-022-05281-z 35505378 PMC9066875

[38] Szczesna J Popiołek M Schmidt K Kowalczyk R. The first record of *Aelurostrongylus abstrusus* (Angistrongylidae: Nematoda) in Eurasian lynx (*Lynx lynx* L.) from Poland based on fecal analysis. Wiad Parazytol. (2006) 52:321–2. 17432626

[39] Jeżewski W Buńkowska-Gawlik K Hildebrand J Perec-Matysiak A Laskowski Z. Intermediate and paratenic hosts in the life cycle of *Aelurostrongylus abstrusus* in natural environment. Vet Parasitol. (2013) 198:401–5. 10.1016/j.vetpar.2013.09.003 24094777

[40] Colella V Knaus M Lai O Cantile C Abramo F Rehbein S. Mice as paratenic hosts of *Aelurostrongylus abstrusus*. Parasites Vectors. (2019) 12:49. 10.1186/s13071-019-3293-2 30670072 PMC6343334

[41] Deplazes P Joachim A Mathis A Strube C Taubert A Samson-Himmelstjerna G von. Parasitologie für die Tiermedizin. 4., überarbeitete Auflage. Stuttgart New York: Georg Thieme Verlag (2021). p. 687. 10.1055/b-0040-179249

[42] Bezerra-Santos MA Mendoza-Roldan JA Abramo F Lia RP Tarallo VD Salant H. Transmammary transmission of *Troglostrongylus brevior* feline lungworm: a lesson from our gardens. Vet Parasitol. (2020) 285:109215. 10.1016/j.vetpar.2020.109215 32862125 PMC7428694

[43] Brianti E Giannetto S Dantas-Torres F Otranto D. Lungworms of the genus *Troglostrongylus* (Strongylida: Crenosomatidae): Neglected parasites for domestic cats. Vet Parasitol. (2014) 202:104–12. 10.1016/j.vetpar.2014.01.019 24566126

[44] Morelli S Diakou A Colombo M Di Cesare A Barlaam A Dimzas D. Cat respiratory nematodes: current knowledge, novel data and warranted studies on clinical features, treatment and control. Pathogens. (2021) 10:454. 10.3390/pathogens10040454 33920104 PMC8069686

[45] Biocca E. *Angiostrongylus chaubdi* n. sp parassita del cuore e dei vasi polmonari del gatto selvatico (*Felis silvestris*) R. Accad Naz Lincei. (1957) 22:526–32.

[46] Giannelli A Colella V Abramo F. do Nascimento Ramos RA, Falsone L, Brianti E, et al. Release of lungworm larvae from snails in the environment: potential for alternative transmission pathways Knight M, editor. PLoS Negl Trop Dis. (2015) 9:e0003722. 10.1371/journal.pntd.0003722 25884402 PMC4401693

[47] Brianti E Gaglio G Napoli E Falsone L Giannetto S Latrofa MS. Evidence for direct transmission of the cat lungworm *Troglostrongylus brevior* (Strongylida: Crenosomatidae). Parasitology. (2013) 140:821–4. 10.1017/S0031182013000188 23552474

[48] Diakou A Di Cesare A Aeriniotaki T Traversa D. First report of *Troglostrongylus brevior* in a kitten in Greece. Parasitol Res. (2014) 113:3895–8. 10.1007/s00436-014-4122-3 25195058

[49] Falsone L Brianti E Gaglio G Napoli E Anile S Mallia E. The European wildcats (*Felis silvestris silvestris*) as reservoir hosts of *Troglostrongylus brevior* (Strongylida: Crenosomatidae) lungworms. Vet Parasitol. (2014) 205:193–8. 10.1016/j.vetpar.2014.06.024 25027610

[50] Traversa D Lepri E Veronesi F Paoletti B Simonato G Diaferia M. Metastrongyloid infection by *Aelurostrongylus abstrusus, Troglostrongylus brevior* and *Angiostrongylus chabaudi* in a domestic cat. Int J Parasitol. (2015) 45:685–90. 10.1016/j.ijpara.2015.05.005 26149643

[51] Cavalera MA Iatta R Colella V Dantas-Torres F Corsaro A Brianti E. *Troglostrongylus brevior*: a feline lungworm of paediatric concern. Vet Parasitol. (2018) 253:8–11. 10.1016/j.vetpar.2018.02.017 29605009

[52] Segeritz L Westhoff KM Schaper R Hermosilla C Taubert A. *Angiostrongylus vasorum, Aelurostrongylus abstrusus*, *Crenosoma vulpis* and *Troglostrongylus brevior* infections in native slug populations of Bavaria and Baden-Wuerttemberg in Germany. Pathogens. (2022) 11:747. 10.3390/pathogens11070747 35889992 PMC9315663

[53] Veronesi F Traversa D Lepri E Morganti G Vercillo F Grelli D. Occurrence of lungworms in European wildcats (*Felis silvestris silvestris*) of central Italy. J Wildl Dis. (2016) 52:270–8. 10.7589/2015-07-187 26967134

[54] Reichard MV Caudell DL Alan Kocan A. Survey of helminth lung parasites of bobcats (*Lynx rufus*) from Alabama, Kansas, New Mexico, Oklahoma, and Virginia, USA. Compar Parasitol. (2004) 71:88–90. 10.1654/4086

[55] Penagos-Tabares F Lange MK Vélez J Hirzmann J Gutiérrez-Arboleda J Taubert A. The invasive giant African snail *Lissachatina fulica* as natural intermediate host of *Aelurostrongylus abstrusus, Angiostrongylus vasorum, Troglostrongylus brevior*, and *Crenosoma vulpis* in Colombia. PLoS Negl Trop Dis. (2019) 13:e0007277. 10.1371/journal.pntd.0007277 31002674 PMC6493767

[56] Pun K Albrecht C Castella V Fumagalli L. Species identification in mammals from mixed biological samples based on mitochondrial DNA control region length polymorphism. Electrophoresis. (2009) 30:1008–14. 10.1002/elps.200800365 19229844

[57] Eckert I Suchentrunk F Markov G Hartl GB. Genetic diversity and integrity of German wildcat (*Felis silvestris*) populations as revealed by microsatellites, allozymes, and mitochondrial DNA sequences. Mammalian Biology. (2010) 75:160–74. 10.1016/j.mambio.2009.07.005

[58] Fumagalli L Taberlet P Favre L Hausser J. Origin and evolution of homologous repeated sequences in the mitochondrial DNA control region of shrews. Mol Biol Evol. (1996) 13:31–46. 10.1093/oxfordjournals.molbev.a025568 8583904

[59] Altschul SF Gish W Miller W Myers EW Lipman DJ. Basic local alignment search tool. J Molec Biol. (1990) 215:403–410. 10.1016/S0022-2836(05)80360-2 2231712

[60] Baermann G. Eine einfache Methode zur Auffindung von Ankylostomum (Nematoden) Larven. Geneeskundig Tijdschrift Voor Nederlandsch-Indië. (1916) 57:131–7.

[61] Thienpont D Rochette F Vanparijs OFJ. Diagnosing Helminthiasis by Coprological Examination. Beerse: Janssen Animal Health (2003).

[62] George MM Lopez-Soberal L Storey BE Howell SB Kaplan RM. Motility in the L3 stage is a poor phenotype for detecting and measuring resistance to avermectin/milbemycin drugs in gastrointestinal nematodes of livestock. Int J Parasitol. (2018) 8:22–30. 10.1016/j.ijpddr.2017.12.002 29274827 PMC6114081

[63] Scott DW. Current Knowledge of Aelurostrongylosis in the cat - Literature review and case reports. Cornell Vet. (1973) 63:483–500. 4782565

[64] Giannelli A Kirkova Z Abramo F Latrofa MS Campbell B Zizzo N. *Angiostrongylus chabaudi* in felids: new findings and a review of the literature. Vet Parasitol. (2016) 228:188–92. 10.1016/j.vetpar.2016.09.007 27692325

[65] Gasser RB Chilton NB Hoste H Beveridge I. Rapid sequencing of rDNA from single worms and eggs of parasitic helminths. Nucl Acids Res. (1993) 21:2525–6. 10.1093/nar/21.10.2525 8506152 PMC309567

[66] Annoscia G Latrofa MS Campbell BE Giannelli A Ramos RAN Dantas-Torres F. Simultaneous detection of the feline lungworms *Troglostrongylus brevior* and *Aelurostrongylus abstrusus* by a newly developed duplex-PCR. Vet Parasitol. (2014) 199:172–8. 10.1016/j.vetpar.2013.10.015 24238839

[67] Conboy GA Addams C. Treatment of Crenosoma vulpis infection in two silver foxes (*Vulpes vulpes*) with ivermectin. J Zoo Wildlife Med. (1995) 26:597–600.

[68] Barutzki D Laubmeier E Forstner MJ. Endoparasitic infestation of wild hedgehogs and hedgehogs in human care with a contribution to therapy. Tierärztliche Praxis. (1987) 15:325–31. 3424361

[69] Campbell WC. Ivermectin and Abamectin. New York, NY: Springer. (1994).

[70] Kirkpatrick CE Megella C. Use of ivermectin in treatment of Aelurostrongylus abstrusus and Toxocara cati infections in a cat. J Am Vet Med Assoc. (1987) 190:1309–10. 10.2460/javma.1987.190.10.1309 3583886

[71] Szczesna J Popiołek M Schmidt K Kowalczyk R. Coprological study on helminth fauna in eurasian lynx (*Lynx lynx*) from the białowieża primeval forest in Eastern Poland. J Parasitol. (2008) 94:981–4. 10.1645/GE-1440.1 18576790

[72] Tintori SC Sloat SA Rockman MV. Rapid Isolation of Wild Nematodes by Baermann Funnel. JoVE. (2022) (179):63287. 10.3791/63287-v 35156660 PMC8857960

[73] Snyder PW Hogg JT Ezenwa VO. Comparison of modified Flotac and Baermann techniques for quantifying lungworm larvae in free-ranging bighorn sheep (*Ovis canadensis*) feces, Montana, USA. J Wildl Dis. (2015) 51:843–8. 10.7589/2014-10-244 26267464

[74] Barutzki D Schaper R. Natural Infections of *Angiostrongylus vasorum* and *Crenosoma vulpis* in Dogs in Germany (2007–2009). Parasitol Res. (2009) 105:39–48. 10.1007/s00436-009-1494-x 19575224

[75] Lopez-Osorio S Navarro-Ruiz JL Rave A Taubert A Hermosilla C Chaparro-Gutierrez JJ. *Aelurostrongylus abstrusus* infections in domestic cats (*Felis silvestris catus*) from Antioquia, Colombia. Pathogens. (2021) 10:337. 10.3390/pathogens10030337 33805839 PMC7998092

[76] Wulcan JM Timmins A Dennis MM Thrall MA Lejeune M Abdu A. First report of *Aelurostrongylus abstrusus* in St. Kitts Veter Parasitol. (2020) 19:100366. 10.1016/j.vprsr.2019.100366 32057393

[77] Penagos-Tabares F Lange MK Chaparro-Gutiérrez JJ Taubert A Hermosilla C. *Angiostrongylus vasorum* and *Aelurostrongylus abstrusus*: neglected and underestimated parasites in South America. Parasites Vectors. (2018) 11:208. 10.1186/s13071-018-2765-0 29587811 PMC5870519

[78] Traversa D Guglielmini C. Feline aelurostrongylosis and canine angiostrongylosis: a challenging diagnosis for two emerging verminous pneumonia infections. Vet Parasitol. (2008) 157:163–74. 10.1016/j.vetpar.2008.07.020 18775603

[79] Lacorcia L Gasser RB Anderson GA Beveridge I. Comparison of bronchoalveolar lavage fluid examination and other diagnostic techniques with the Baermann technique for detection of naturally occurring *Aelurostrongylus abstrusus* infection in cats. JAVMA. (2009) 235:43–9. 10.2460/javma.235.1.43 19566453

[80] Valdmann H Moks E Talvik H. Helminth fauna of Eurasian lynx (*Lynx lynx*) in Estonia. J Wildl Dis. (2004) 40:356–60. 10.7589/0090-3558-40.2.356 15362842

[81] Deksne G Laakkonen J Näreaho A Jokelainen P Holmala K Kojola I. Endoparasites of the Eurasian Lynx (*Lynx lynx*) in Finland. J Parasitol. (2013) 99:229–34. 10.1645/GE-3161.1 23016871

[82] Vezzosi T Perrucci S Parisi F Morelli S Maestrini M Mennuni G. Fatal pulmonary hypertension and right-sided congestive heart failure in a kitten infected with *Aelurostrongylus abstrusus*. Animals. (2020) 10:2263. 10.3390/ani10122263 33271887 PMC7759851

[83] Philbey AW Krause S Jefferies R. Verminous pneumonia and enteritis due to hyperinfection with *Aelurostrongylus abstrusus* in a kitten. J Comp Pathol. (2014) 150:357–60. 10.1016/j.jcpa.2014.02.001 24679855

[84] Crisi PE Traversa D Di Cesare A Luciani A Civitella C Santori D. Irreversible pulmonary hypertension associated with *Troglostrongylus brevior* infection in a kitten. Res Vet Sci. (2015) 102:223–7. 10.1016/j.rvsc.2015.08.019 26412548

[85] Smith JD Addison EM Joachim DG Smith LM Quinn NWS. Helminth parasites of Canada lynx (*Felis canadensis*) from northern Ontario. Can J Zool. (1986) 64:358–64. 10.1139/z86-057

[86] Klewer HL. The incidence of helminth lung parasites of *Lynx rufus rufus* (Schreber) and the life cycle of *Anfilaroides rostratus*. J Parasitol. (1958) 44:1516.

[87] Little JW Smith JP Knowlton FF Bell RR. Incidence and geographic distribution of some nematodes in texas bobcats. Tex J Sci. (1971) 22:403–7.

[88] Watson TG Nettles VF Davidson WR. Endoparasites and selected infectious agents in bobcats (*Felis rufus*) from West Virgina and Georgia. J Wildl Dis. (1981) 17:547–544. 10.7589/0090-3558-17.4.547 6802988

[89] Schmidt-Posthaus H Breitenmoser-Wörsten C Posthaus H Bacciarini L Breitenmoser U. Causes of mortality in reintroduced Eurasian lynx in Switzerland. J Wildl Dis. (2002) 38:84–92. 10.7589/0090-3558-38.1.84 11838233

[90] Otranto D Brianti E Dantas-Torres F. *Troglostrongylus brevior* and a nonexistent ‘dilemma'. Trends Parasitol. (2013) 29:517–8. 10.1016/j.pt.2013.09.001 24080064

[91] Giannelli A Passantino G Ramos RAN Lo Presti G Lia RP Brianti E. Pathological and histological findings associated with the feline lungworm *Troglostrongylus brevior*. Vet Parasitol. (2014) 204:416–9. 10.1016/j.vetpar.2014.05.020 24912956

[92] Brianti E Gaglio G Giannetto S Annoscia G Latrofa MS Dantas-Torres F. *Troglostrongylus brevior* and *Troglostrongylus subcrenatus* (*Strongylida: Crenosomatidae*) as agents of broncho-pulmonary infestation in domestic cats. Parasites Vectors. (2012) 5:178. 10.1186/1756-3305-5-178 22916686 PMC3469345

[93] Schug K Krämer F Schaper R Hirzmann J Failing K Hermosilla C. Prevalence survey on lungworm (*Angiostrongylus vasorum, Crenosoma vulpis, Eucoleus aerophilus*) infections of wild red foxes (*Vulpes vulpes*) in central Germany. Parasites Vectors. (2018) 11:85. 10.1186/s13071-018-2672-4 29409523 PMC5801722

[94] Nonnis F Tamponi C Tosciri G Manconi M Pudda F Cabras P. Cardio-pulmonary nematodes of the red fox (*Vulpes vulpes*) of Sardinia, Italy. Parasitol Res. (2023) 122:1685–8. 10.1007/s00436-023-07882-8 37212835 PMC10276099

[95] Odden J Linnell JDC Andersen R. Diet of Eurasian lynx, Lynx lynx, in the boreal forest of southeastern Norway: the relative importance of livestock and hares at low roe deer density. Eur J Wildl Res. (2006) 52:237–44. 10.1007/s10344-006-0052-4

[96] Krofel M Huber D Kos I. Diet of Eurasian lynx Lynx lynx in the northern Dinaric Mountains (Slovenia and Croatia): Importance of edible dormouse Glis glis as alternative prey. Acta Theriol. (2011) 56:315–22. 10.1007/s13364-011-0032-2

[97] Mariacher A Santini A Del Lesto I Tonon S Cardini E Barone A. Endoparasite infections of the European hedgehog (*Erinaceus europaeus*) in Central Italy. Animals. (2021) 11:3171. 10.3390/ani11113171 34827903 PMC8614308

[98] Anders O Kaphegyi TAM Kubik F. Untersuchungen zum Dispersionsverhalten eines männlichen Luchses (Lynx lynx) im Dreiländereck zwischen Thüringen, Niedersachsen und Hessen. Säugetierkundliche Informationen, Jena. (2012) 45:455–62.

[99] Mayer K Belotti E Bufka L Heurich M. Dietary patterns of the Eurasian lynx (Lynx lynx) in the Boehmian forest. Säugetierkundliche Informationen, Jena. (2012) 45:447–453.

[100] Millán J Casanova JC. Helminth parasites of the endangered Iberian lynx (*Lynx pardinus*) and sympatric carnivores. J Helminthol. (2007) 81:377–80. 10.1017/S0022149X07869203 18021466

[101] León CI García-Bocanegra I McCain E Rodríguez E Zorrilla I Gómez AM. Prevalence of selected pathogens in small carnivores in reintroduction areas of the Iberian lynx (*Lynx pardinus*). Veterinary Record. (2017) 180:252–252. 10.1136/vr.104038 28062843

[102] Diakou A Migli D Dimzas D Morelli S Di Cesare A Youlatos D. Endoparasites of European wildcats (*Felis silvestris*) in Greece. Pathogens. (2021) 10:594. 10.3390/pathogens10050594 34068209 PMC8153176

[103] Colella V Cavalera MA Deak G Tarallo VD Gherman CM Mihalca AD. Larval development of *Angiostrongylus chabaudi*, the causative agent of feline angiostrongylosis, in the snail *Cornu aspersum*. Parasitology. (2017) 144:1922–30. 10.1017/S0031182017001433 28805181

[104] Traversa D Di Cesare A. Diagnosis and management of lungworm infections in cats: cornerstones, dilemmas and new avenues. J Feline Med Surg. (2016) 18:7–20. 10.1177/1098612X15623113 26733545 PMC11148874

[105] Wiese V. Die Landschnecken Deutschlands, Finden - Erkennen - Bestimmen. 2 Auflage Wiebelsheim: Quelle & Meyer Verlag GmbH & Co. (2016).

[106] Kerney MP Cameron RobertAD Jungbluth JH. Die Landschnecken Nord- und Mitteleuropas - Ein Bestimmungsbuch für Biologen und Naturfreunde. 1. Auflage. Hamburg und Berlin: Paul Parey (1983). p. 384.

[107] Kim JR Hayes KA Yeung NW Cowie RH. Diverse Gastropod Hosts of *Angiostrongylus cantonensis*, the Rat Lungworm, globally and with a focus on the Hawaiian Islands. PLoS ONE. (2014) 9:e94969. 10.1371/journal.pone.0094969 24788772 PMC4008484

[108] Medeiros MCI Rollins RL Echaluse MV Cowie RH. Species identity and size are associated with rat lungworm infection in gastropods. Ecohealth. (2020) 17:183–93. 10.1007/s10393-020-01484-x 32676832

[109] Segeritz L Cardona A Taubert A Hermosilla C Ruiz A. Autochthonous *Angiostrongylus cantonensis, Angiostrongylus vasorum* and *Aelurostrongylus abstrusus* infections in native terrestrial gastropods from the Macaronesian Archipelago of Spain. Parasitol Res. (2021) 120:2671–80. 10.1007/s00436-021-07203-x 34180003 PMC8263545

[110] Aziz NAA Daly E Allen S Rowson B Greig C Forman D. Distribution of *Angiostrongylus vasorum* and its gastropod intermediate hosts along the rural–urban gradient in two cities in the United Kingdom, using real time PCR. Parasites Vectors. (2016) 9:56. 10.1186/s13071-016-1338-3 26830203 PMC4736697

[111] Gueldner EK Schuppisser C Borel N Hilbe M Schnyder M. First case of a natural infection in a domestic cat (*Felis catus*) with the canid heart worm *Angiostrongylus vasorum*. Veter Parasitol. (2019) 18:100342. 10.1016/j.vprsr.2019.100342 31796174 PMC7104072

[112] Di Cesare A Morelli S Colombo M Simonato G Veronesi F Marcer F. Is angiostrongylosis a realistic threat for domestic cats? Front Vet Sci. (2020) 7:195. 10.3389/fvets.2020.00195 32351980 PMC7174740

[113] Härtwig V Schulze C Barutzki D Schaper R Daugschies A Dyachenko V. Detection of *Angiostrongylus vasorum* in red foxes (*Vulpes vulpes*) from Brandenburg, Germany. Parasitol Res. (2015) 114:185–92. 10.1007/s00436-015-4524-x 26152419

[114] Colella V Giannelli A Brianti E Ramos RAN Cantacessi C Dantas-Torres F. Feline lungworms unlock a novel mode of parasite transmission. Sci Rep. (2015) 5:13105. 10.1038/srep13105 26271902 PMC4536521

[115] Modrý D Fecková B Putnová B Manalo SM Otranto D. Alternative pathways in *Angiostrongylus cantonensis* (Metastrongyloidea: Angiostrongylidae) transmission. Parasitology. (2021) 148:167–73. 10.1017/S0031182020001857 32981541 PMC11010052

